# Long-term heat-storage ceramics absorbing thermal energy from hot water

**DOI:** 10.1126/sciadv.aaz5264

**Published:** 2020-07-01

**Authors:** Yoshitaka Nakamura, Yuki Sakai, Masaki Azuma, Shin-ichi Ohkoshi

**Affiliations:** 1 Industrial Solutions Company, Panasonic Corporation, 1006 Kadoma, Kadoma City, Osaka 571-8501, Japan.; 2 Kanagawa Institute of Industrial Science and Technology 705-1 Shimoimaizumi, Ebina, Kanagawa 243-0435, Japan.; 3 Laboratory for Materials and Structures, Tokyo Institute of Technology, 4259 Nagatsuta, Midori-ku, Yokohama, Kanagawa 226-8503, Japan.; 4 Department of Chemistry, School of Science, The University of Tokyo, 7-3-1 Hongo, Bunkyo-ku, Tokyo 113-0033, Japan.

## Abstract

In thermal and nuclear power plants, 70% of the generated thermal energy is lost as waste heat. The temperature of the waste heat is below the boiling temperature of water. Here, we show a long-term heat-storage material that absorbs heat energy at warm temperatures from 38°C (311 K) to 67°C (340 K). This unique series of material is composed of scandium-substituted lambda-trititanium-pentoxide (λ-Sc*_x_*Ti_3−*x*_O_5_). λ-Sc*_x_*Ti_3−*x*_O_5_ not only accumulates heat energy from hot water but also could release the accumulated heat energy by the application of pressure. λ-Sc*_x_*Ti_3−*x*_O_5_ has the potential to accumulate heat energy of hot water generated in thermal and nuclear power plants and to recycle the accumulated heat energy on demand by applying external pressure. Furthermore, it may be used to recycle waste heat in industrial factories and automobiles.

## Teaser

Heat energy bank: Accumulated heat energy is eternally preserved and extracted on demand by pressure.

## INTRODUCTION

Generated thermal energy cannot be efficiently converted to electric power at thermal and nuclear power plants. Seventy percent of the generated thermal energy is discarded as waste heat (*1*–*4*). The temperature of this waste heat is below the boiling temperature of water, i.e., 100°C (373 K) (*5*). The waste heat is currently released into the atmosphere through water or air, negatively affecting the environment (*6*–*12*). Storing and using this waste heat would provide numerous benefits due to the improved energy efficiency and environmental compliance. In the present paper, we report a long-term heat-storage ceramic, scandium-substituted lambda-trititanium-pentoxide, absorbing thermal energy by a solid-solid phase transition below boiling temperature of water. The ceramic can repeatedly use thermal energy by pressure and heating. This heat-storage performance could provide a sophisticated energy reuse technology for thermal and nuclear power plants and mitigate negative environmental impact of the waste heat.

## RESULTS

### First-principles calculations of formation energy

**Fig. 1:**
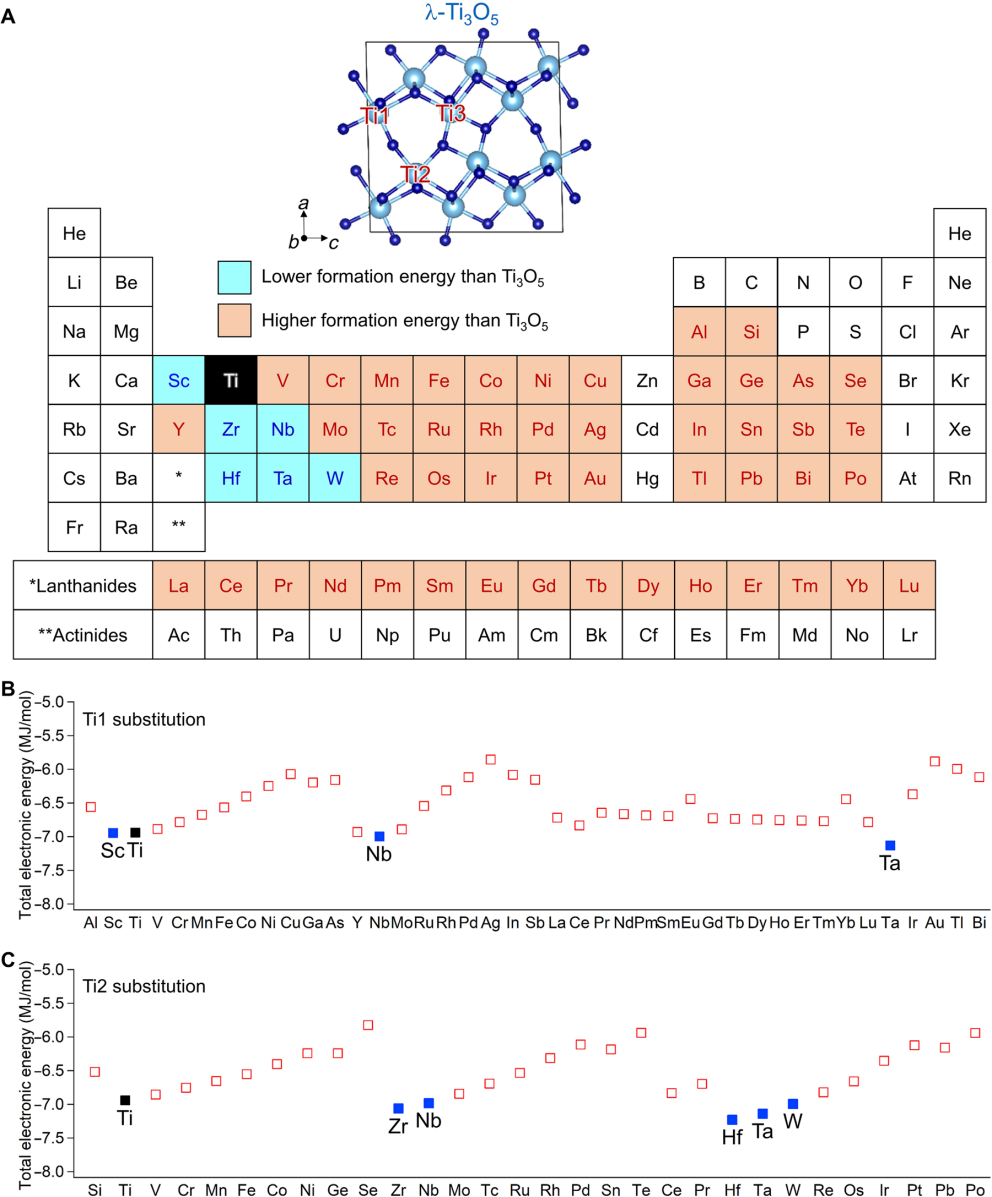
First-principles calculations of formation energies. (**A**) Periodic table colored by the total electronic energies of λ-Ti_3_O_5_ with an elemental substitution. Blue elements are those where substituted λ-Ti_3_O_5_ shows a lower formation energy than that of pure λ-Ti_3_O_5_. Orange elements are those where substituted λ-Ti_3_O_5_ shows a higher formation energy. (**B**) Calculated total electronic energies of λ-A*_x_*Ti_3−*x*_O_5_ (A, trivalent elements) and (**C**) λ-B*_x_*Ti_3-*x*_O_5_ (B, tetravalent elements) in order of atomic number. One of three Ti sites in λ-Ti_3_O_5_ is substituted by a colored element for the first-principles calculations. Element A in λ-A*_x_*Ti_3−*x*_O_5_ substitutes into the Ti1 site. Element B in λ-B*_x_*Ti_3-*x*_O_5_ substitutes into the Ti2 site. Blue and orange squares represent that elemental substituted λ-Ti_3_O_5_ shows a lower formation and a higher formation energy, respectively. Black square denotes pure λ-Ti_3_O_5_.

In an effort to realize heat-storage materials (*13*, *14*) capable of absorbing low-temperature waste heat, our research has focused on metal-substituted lambda-trititanium-pentoxide (λ-*M_x_*Ti_3_O_5_). λ-Ti_3_O_5_ exhibits photo- and pressure-induced phase transitions (*15*–*19*). To date, several types of metal-substituted λ-Ti_3_O_5_ have been reported (*20*–*22*). We surveyed metal cations suitable for metal substitution of the Ti ion in λ-Ti_3_O_5_. Specifically, we conducted first-principles calculations and determined the formation energies of the various λ-*M_x_*Ti_3_O_5_ using 54 different elements. Figure 1A and fig. S1 show the results, where blue denotes that metallic ion substitution stabilizes the formation energy, while orange destabilizes the formation energy.

Of these elements, only six have a stabilizing effect: Sc, Nb, Ta, Zr, Hf, and W (Fig. 1, B and C). Thus, we synthesized these λ-*M_x_*Ti_3_O_5_. Substituting with Nb, Ta, Zr, Hf, and W yields the β phase. However, Sc-substituted Ti_3_O_5_ assumes the λ phase (fig. S2). Here, we report the synthesis, crystal structure, and heat-storage properties of Sc-substituted λ-Ti_3_O_5_.

### Crystal structure

**Fig. 2:**
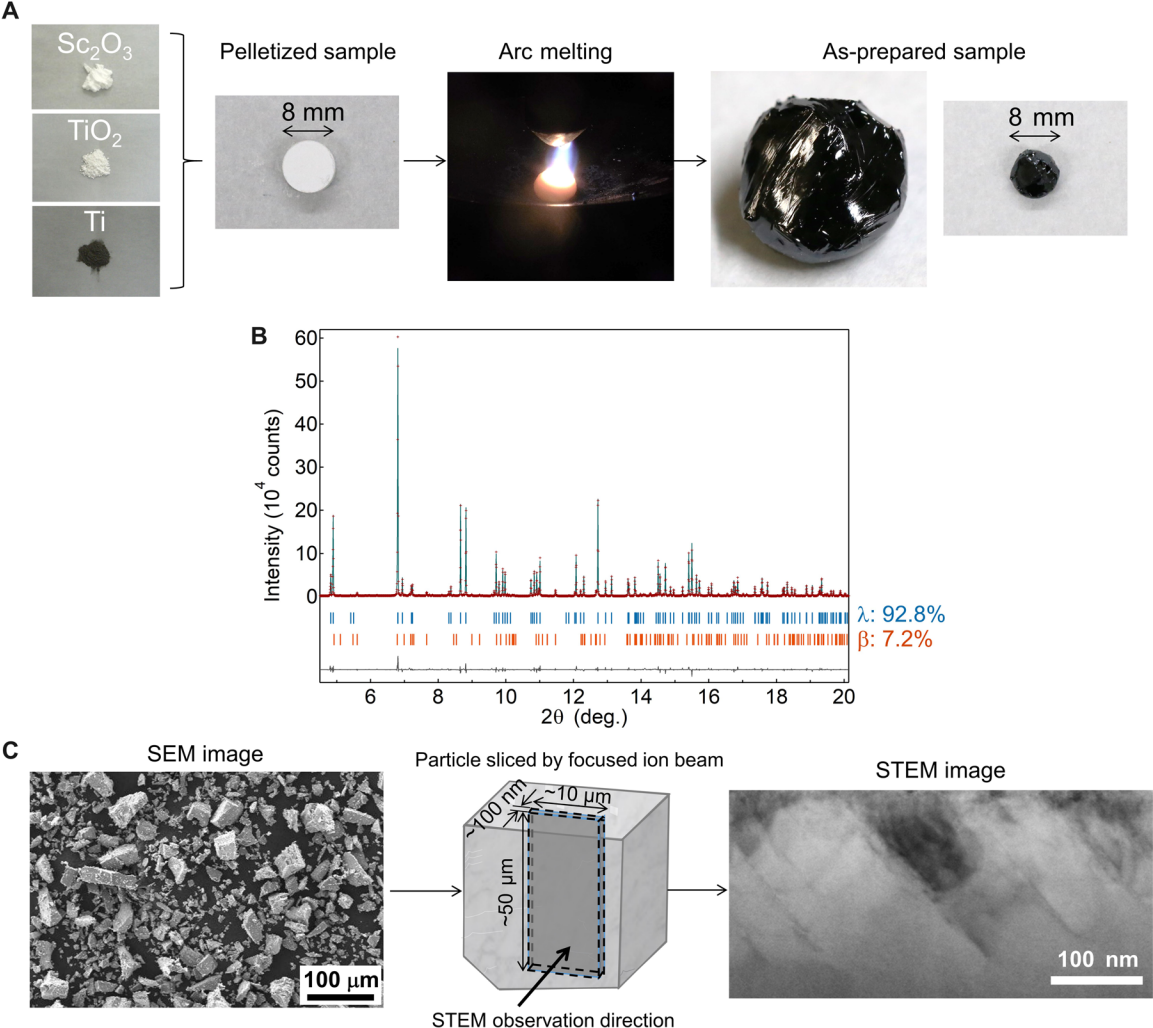
Synthesis, crystal structure, and morphology of λ-Sc_0.09_Ti_2.91_O_5_. (**A**) λ-Sc_0.09_Ti_2.91_O_5_ sample synthesis. Pelletized mixture powder of Sc_2_O_3_, TiO_2_, and Ti metal with a diameter of 8 mm is prepared, melted, and rapidly cooled in an arc-melting process. After the melting process, the solidified (as-prepared) sample is milled by hand. Photo credit: Yoshitaka Nakamura, Panasonic Corporation. (**B**) Synchrotron x-ray diffraction (SXRD) pattern of the as-prepared Sc_0.09_Ti_2.91_O_5_ sample collected at room temperature with λ = 0.420111 Å. Upper blue and lower orange bars represent the calculated positions of the Bragg reflections of λ-Sc_0.09_Ti_2.91_O_5_ and β-Sc_0.09_Ti_2.91_O_5_. (**C**) Scanning electron microscopy (SEM) image of the powdered sample shows a grain size below 100 μm. Particle from the powdered sample is sliced by a focused ion beam. STEM image shows stripe-like domains with a size of about 100 nm × 200 nm. Scale bars show 100 μm in the SEM image and 100 nm in the STEM image.

We used an arc-melting technique to synthesize the Sc-substituted λ-Ti_3_O_5_ (*23*–*27*). Figure 2A overviews the synthetic procedure. Precursors of Sc_2_O_3_, TiO_2_, and Ti powders are mixed, and an 8-mm pellet of the mixture is prepared. Arc melting was used to melt the pellet in an Ar atmosphere. Then, the sample is shaped into a spherical ball (Fig. 2A). The obtained sample is milled by hand. The formula of the sample is determined to be Sc_0.09_Ti_2.91_O_5_ by x-ray fluorescent (XRF) measurements (see Materials and Methods). We performed synchrotron x-ray diffraction (SXRD) measurements using beamline BL02B2 at SPring-8 to determine the crystal structure (*28*). Figure 2B shows the SXRD pattern of the as-prepared sample at room temperature. From the Rietveld analysis, the crystal structure is monoclinic (space group *C*2/*m*) with lattice parameters of *a* = 9.84195 (4) Å, *b* = 3.79151 (1) Å, *c* = 9.98618 (4) Å, β = 91.1207 (3)°, and a unit cell volume of *V* = 372.572 (3) Å^3^ (fig. S3).

These characteristics correspond to the crystal structure of λ-Ti_3_O_5_. λ-Sc_0.09_Ti_2.91_O_5_ has a slightly larger unit cell volume than that of λ-Ti_3_O_5_ with a 0.4% expansion. In addition, the β phase is present as the minor phase. The β phase adopts a monoclinic crystal structure (space group *C*2/*m*) with lattice parameters of *a* = 9.7930 (4) Å, *b* = 3.8064 (14) Å, *c* = 9.4375 (4) Å, β = 91.5611 (3)°, and *V* = 351.66 (2) Å^3^. The scanning transmission electron microscopy (STEM) image shows stripe-like domains measuring approximately 100 nm × 200 nm (Fig. 2C).

### Pressure-induced phase transition

**Fig. 3:**
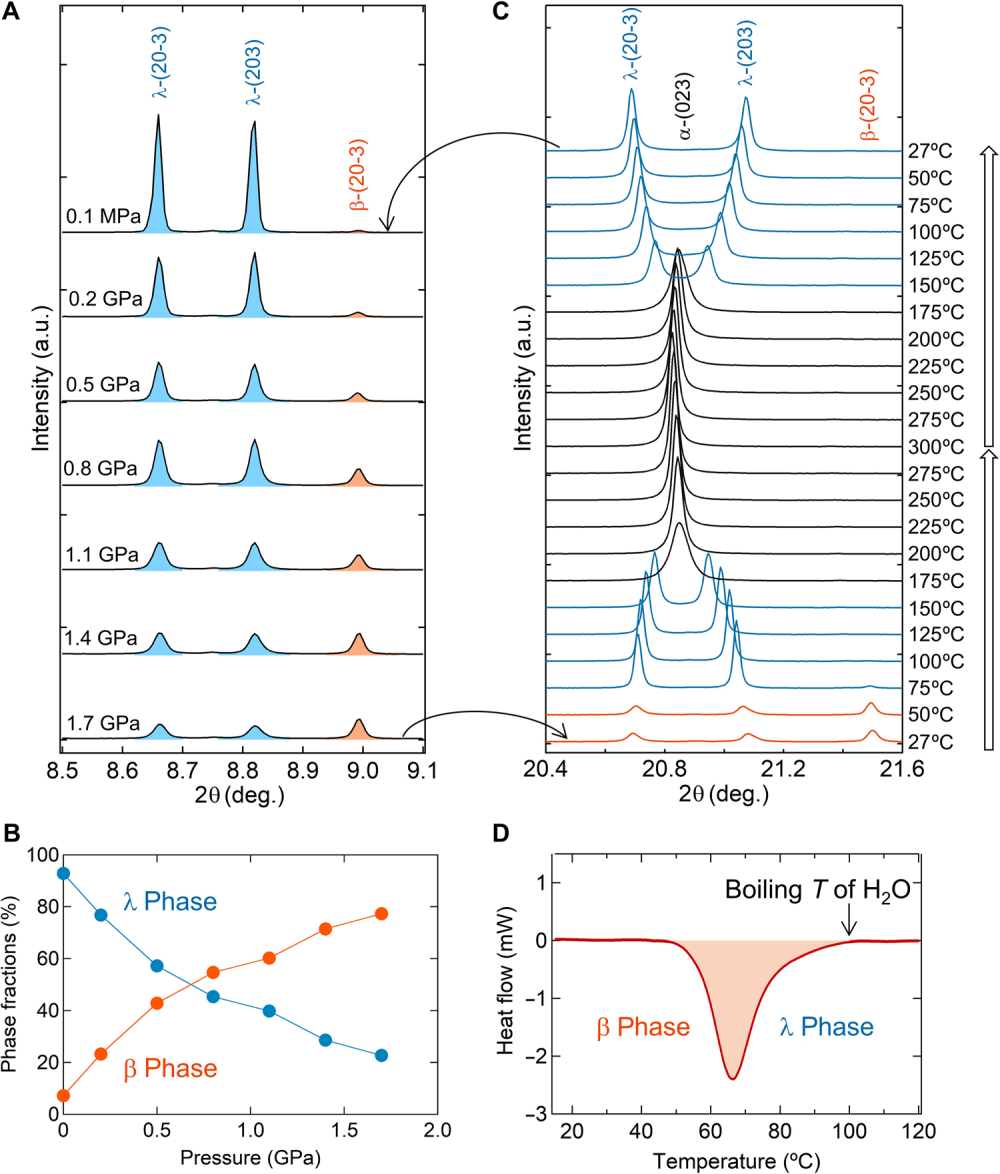
Pressure-induced phase transition and heat-storage process. (**A**) SXRD patterns of Sc_0.09_Ti_2.91_O_5_ measured at room temperature and ambient pressure after compression between 0.2 and 1.7 GPa with a hydraulic press (λ = 0.420111 Å). As the pressure increases, the λ-(20-3) and λ-(203) peaks (blue) decrease and the β-(20-3) peak (orange) increases, indicating a pressure-induced phase transition. a.u., arbitrary units. (**B**) Pressure dependence of the phase fractions of Sc_0.09_Ti_2.91_O_5_ calculated from the SXRD patterns in (A). Crossover pressure (phase transition pressure) occurs at 670 MPa. (**C**) SXRD patterns of Sc_0.09_Ti_2.91_O_5_ measured between 27°C (300 K) and 300°C (573 K; λ = 0.999255 Å). The λ and β peaks are constant until 50°C (323 K; orange), and then the β phase decreases and the λ phase increases at 75°C (348 K; blue). The λ phase transforms into the α phase above 175°C (448 K; black) but is restored upon cooling. (**D**) DSC chart of Sc_0.09_Ti_2.91_O_5_ shows an endothermic reaction at 67°C (340 K). Samples are compressed at 1.7 GPa before the variable temperature SXRD and DSC chart measurements.

Next, we measured the pressure-induced phase transition using SXRD (fig. S4). The as-prepared sample was compressed by pressures of 0.2 to 1.7 GPa with a hydraulic press. As the pressure increases, the λ-phase fraction decreases, while the β-phase fraction increases (Fig. 3A). The crossover pressure is 670 MPa (Fig. 3B). The sample after the pressure-induced phase transition (Fig. 3A) was heated, and the temperature evolution of the SXRD patterns was collected (fig. S5). Figure 3C shows the peaks of λ-(203), λ- and β-(20-3), and α-(023). The λ and β peaks are constant until 50°C (323 K), and then the β phase decreases and the λ phase increases at 75°C (348 K), indicating reversibility due to pressure and heating. The λ phase transforms into the α phase above 175°C (448 K) but, upon cooling, returns to the λ phase in the absence of a transition back to the β phase (fig. S6).

### Heat-storage property

We measured the heat absorption mass of the sample after the pressure-induced phase transition by differential scanning calorimetry (DSC). We swept the sample compressed at 1.7 GPa with 22.7% of the λ phase and 77.3% of the β phase from 0°C (273 K) to 300°C (573 K). Heat absorption is observed with an absorption peak at 67°C (340 K) (Fig. 3D). Considering the conversion of the λ and β phases, the heat absorption mass is 75 kJ liter^−1^. The pressure- and heat-induced phase transitions were repeatedly observed (fig. S7).

**Fig. 4:**
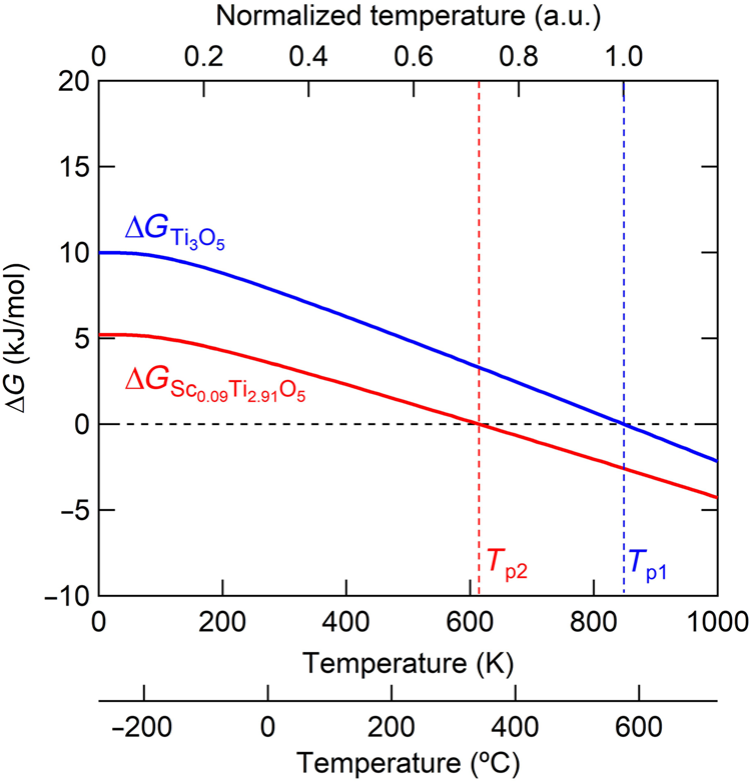
Mechanism for the decrease in transition temperature. Calculated thermodynamic free energy of Ti_3_O_5_ and Sc_0.09_Ti_2.91_O_5_ with supercells (1 × 3 × 1). Sc substitution site for the calculation is set at Ti3, and 1 of 36 Ti atoms is substituted in λ- and β-Ti_3_O_5_. Difference of the free energies of the λ and β phases represents Δ*G* (Δ*G* = *G*_λ_ − *G*_β_). At Δ*G* = 0 (black dotted line), the free energies of the λ and β phases are equal, indicating the crossover temperature (calculated phase transition temperature). Normalized temperature of 1.0 is set at *T*_p1_ = 848 K (575°C), which is the calculated crossover temperature of λ- and β-Ti_3_O_5_ (blue dotted line, Δ*G*_Ti3O5_ = 0). Red dotted line represents the crossover temperature of λ- and β-Sc_0.09_Ti_2.91_O_5_ (Δ*G*_Sc0.09Ti2.91O5_ = 0) at *T*_p2_ = 614 K (341°C). Crossover temperature of Sc_0.09_Ti_2.91_O_5_ decreases about 27.6% from the temperature of Ti_3_O_5_. This temperature decrease ratio agreed well with the experimentally obtained decrease ratio of 27.7% calculated from the phase transition temperature of 470 K (197°C) reported in λ-Ti_3_O_5_ and 340 K (67°C) measured in λ-Sc_0.09_Ti_2.91_O_5_ (Fig. 3D).

Compared to the previous work (*16*), the heat-storage temperature from the pressure-produced β phase to λ phase in the present study is 67°C, which is a remarkable reduction from 197°C. This reduction is attributed to the decrease in the formation energy difference between the two phases, which reduces the crossing temperature of the two Gibbs energy curves (*29*). First-principles calculations support these results. The Gibbs energy versus temperature is described in Fig. 4 and in Materials and Methods.

### Thermodynamic mechanism of long-term heat storage and pressure-induced phase transition

**Fig. 5:**
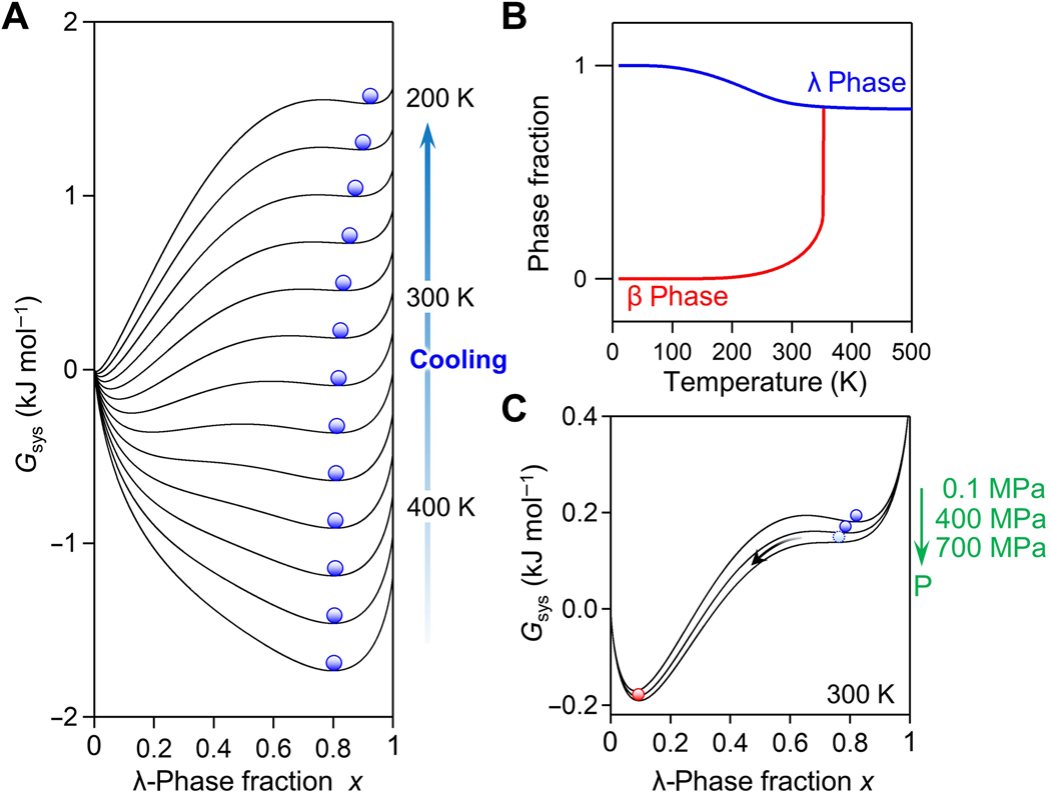
Mechanism of long-term heat storage and pressure-induced phase transition. (**A**) Gibbs free energy (*G*_sys_) versus λ phase fraction (*x*) curves from 420 to 200 K with a 20 K interval, calculated by the SD model. Blue spheres indicate the thermal population of the λ phase. (**B**) Temperature dependence of the calculated λ phase (blue) and β phase (red) fractions. (**C**) *G*_sys_ versus *x* under ambient pressures of 0.1, 400, and 700 MPa at 300 K.

According to previous reports on λ-Ti_3_O_5_ (*15*, *16*), the reversible phase transition between the λ phase and β phase by pressure and heat is considered to be attributed to the energy barrier between the two phases, which originates from the elastic interaction within the material. To understand the mechanisms of long-term heat storage and the low pressure–induced heat energy release, we show the Gibbs free energy of the system (*G*_sys_) using a thermodynamic model based on the Slichter and Drickamer mean-field model (SD model) (*30*) (see Materials and Methods). The Gibbs free energy in the SD model (*G*_sys_) is described as *G*_sys_ *= x*Δ*H* + γ*x*(1 − *x*) + *T*{*R*[*x* ln *x* + (1 − *x*)ln(1 − *x*)] − *x*Δ*S*}, with a cooperative interaction parameter (γ) between the λ phase and β phase due to the elastic interactions within the crystal. *x* is the ratio of λ phase, and *R* is the gas constant. From the result of the DSC measurement, the transition enthalpy (Δ*H*) is 75 kJ liter^−1^ (4.0 kJ mol^−1^), and the transition entropy (Δ*S*) is 0.22 kJ K^−1^ liter^−1^ (12 J K^−1^ mol^−1^). When the interaction parameters are set as a particular combination of values, the SD model calculation well reproduces the measurement data (i.e., the phase transition of β phase → λ phase occurs around 350 K). Then, the thermally produced λ phase is maintained even at low temperatures in the cooling process (Fig. 5, A and B). Thus, the reason why the λ phase is maintained for a long period is that the presence of the energy barrier between the λ and β phases prevents the transformation of the λ phase into the β phase. The prepared λ-Sc_0.09_Ti_2.91_O_5_ shows good stability; i.e., λ-Sc_0.09_Ti_2.91_O_5_ is perfectly maintained after 248 days (about 8 months) and 367 days (1 year) from the XRD measurement.

Furthermore, we reproduced the pressure-induced phase transition from the λ phase to β phase. Applying pressure to the system causes the energy barrier to disappear and induces a phase transition from the λ phase to β phase (Fig. 5C). This pressure-induced phase transition is caused by the change in the γ value upon applying external pressure (see Materials and Methods). Therefore, the system is trapped as the λ phase at room temperature, but applying pressure overcomes the energy barrier, resulting in a phase transition to the β phase.

## DISCUSSION

**Fig. 6:**
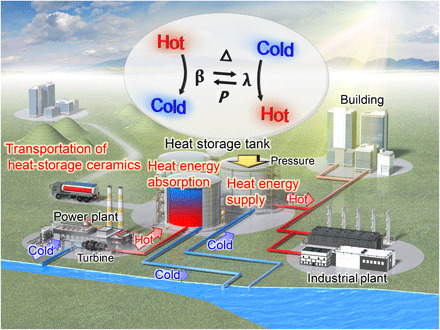
Application of Sc-substituted λ-Ti_3_O_5_ for power plants. Schematic illustration of a heat energy recycling system using Sc-substituted λ-Ti_3_O_5_ heat-storage ceramics. Cooling water for a turbine in a power plant is pumped from a river or sea. Water becomes hot after heat exchange through the turbine. This hot water energy is stored in tanks containing Sc-substituted λ-Ti_3_O_5_ heat-storage ceramics. Water with a reduced heat energy returns to the river or the sea, mitigating the rise of the sea temperature. Energy-stored Sc-substituted λ-Ti_3_O_5_ heat-storage ceramics can supply heat energy to buildings or industrial plants by applying pressure. Furthermore, the energy-stored ceramics can be transported to distant locations by a truck.

Figure 6 schematically illustrates the heat-storage system using Sc-substituted λ-Ti_3_O_5_. Cooling water for a turbine in a power plant is pumped from a river or sea. As the water passes through the turbine, the water temperature increases due to heat exchange. The energy of hot water is transferred to Sc-substituted λ-Ti_3_O_5_ in tanks. Subsequently, water with a reduced thermal energy returns to the river or the sea. This system can mitigate the rise of river or sea water temperature. Energy-stored Sc-substituted λ-Ti_3_O_5_ can release its stored thermal energy by application of pressure, allowing energy to be used on demand. For example, the stored thermal energy can be supplied to buildings or industrial plants that are close to power plants, without using electricity. Moreover, taking advantage of the characteristic of holding the latent heat energy until pressure application, if energy-stored Sc-substituted λ-Ti_3_O_5_ is transported by truck, the heat energy can be used at a distant location. As for the efficiency, the transformation energy efficiency (*e*) value is evaluated on the basis of the temperature dependence of the enthalpy for the λ phase and β phase obtained by first-principles calculations and DSC measurements (fig. S8). For example, when the heat release temperature is 15°C (288 K) and the temperature increase is 1 K, the efficiency is 93%. When the temperature increase is 5 K, the efficiency is 77% (table S1).

In conclusion, we demonstrate heat-storage ceramics based on Sc-substituted λ-Ti_3_O_5_, which absorb heat from hot water. After conducting first-principles calculations, we synthesize Sc-substituted λ-Ti_3_O_5_ ceramics with a heat absorption below 100°C (373 K). This heat absorption material below 100°C can recover the thermal energy from cooling water in power plant turbines, mitigating the rise in sea water temperatures. Moreover, the heat absorption temperature can be easily controlled by changing the Sc content in λ-Ti_3_O_5_ in accordance to the target application.

These heat absorption temperature changes are attributed to the crossover temperature change of Gibbs energies. We successfully synthesize λ-Sc_0.105_Ti_2.895_O_5_ with a heat absorption temperature at 45°C (318 K) and λ-Sc_0.108_Ti_2.892_O_5_ with a heat absorption temperature at 38°C (311 K; see Materials and Methods and fig. S9). Sc-substituted λ-Ti_3_O_5_ will expand opportunities to use thermal energy as it can use thermal energy that is currently in the unused temperature range. In addition to electric power plants, other applications of the present material such as heat-storage usage to collect waste heat from factories, transportation vehicles, mobile phones, and electronic devices should be possible.

## MATERIALS AND METHODS

### First-principles calculations

In consideration of the valences between six-coordinated Ti^3+^ and Ti^4+^ in λ-Ti_3_O_5_ (*15*, *16*, *31*), the total electronic energies of λ-Ti_3_O_5_ substituted by trivalent or tetravalent elements from one of three Ti sites were calculated by first-principles calculations using the Vienna ab initio simulation package (VASP) code. The crystal structure of λ-Ti_3_O_5_ shown in (*16*) was used as calculation models for the initial structure. The lattice parameters and atomic positions were optimized at standard pressure with a cutoff energy of 500 eV and a *k*-mesh of 7 × 7 × 2 until the electronic iterations converged below 10^−5^ eV. On the basis of first-principles calculations, we focused on the synthesis of Sc-substituted Ti_3_O_5_ because Sc takes Sc^3+^ with a six- or eight-coordinated geometry, which hinders the higher valence states in Ti sites observed in β-Ti_3_O_5_.

### Material synthesis by arc melting

Sc-substituted λ-Ti_3_O_5_ samples were synthesized by an arc-melting method with a pelletized mixture powder of Ti metal (99.9% purity), TiO_2_ (99.9% purity), and Sc_2_O_3_ (99.99% purity) in an Ar atmosphere at 0.05 MPa. Ti metal, TiO_2_, and Sc_2_O_3_ powders were mixed in a molar ratio of Ti:TiO_2_:Sc_2_O_3_ = 0.478:2.433:0.045. In this method, samples were melted and turned over three or four times on a copper cooling stage after solidification. These solidified samples were milled by hand before the measurements. The composition was confirmed by XRF, and the formula was determined to be Sc_0.09_Ti_2.91_O_5_: calcd: Ti, 62.4; O, 35.8; Sc, 1.8%; found: Ti, 62.0; O, 36.0; Sc, 2.0%, which is identical to the mixed ratio of the starting materials. Sc_0.105_Ti_2.895_O_5_ and Sc_0.108_Ti_2.892_O_5_ samples were also synthesized, and we measured the x-ray diffraction patterns (fig. S9). Moreover, Ti_3_O_5_ samples, substituted by 3 atomic % (at %) of Zr, Nb, Hf, Ta, and W, were synthesized and their x-ray diffraction patterns were measured (fig. S2). They mainly showed β-Ti_3_O_5_ patterns.

### SXRD measurement

Crystal structures of Sc-substituted Ti_3_O_5_ samples were determined by Rietveld analysis of the SXRD data collected in beamline BL02B2 at SPring-8 (*28*). The samples were sealed in glass capillaries for the SXRD measurements. The RIETAN-FP program was used to refine the structural parameters (*32*).

### Thermal property measurement

The heat absorption properties of Sc-substituted Ti_3_O_5_ samples were measured by DSC (Seiko Instruments, DSC 220C) at a heating-cooling rate of 10 K/min and an air gas flow of 100 ml/min. Before DSC measurements, the samples containing both the λ phase and the β phase were compressed at 1.7 GPa to transform them from the λ phase to the β phase. In addition, the thermal properties of λ-Sc_0.105_Ti_2.895_O_5_ and λ-Sc_0.108_Ti_2.892_O_5_ samples were measured (fig. S9).

### First-principles calculation of Gibbs free energy

To interpret the phase transition temperature, the Gibbs free energies of Sc-substituted λ-Ti_3_O_5_ and β-Ti_3_O_5_ with supercells (1 × 3 × 1) and a *k*-mesh of 2 × 2 × 2 of the optimized structures were calculated using the Phonopy code in cooperation with the VASP code for the interatomic force constants calculations (*33*, *34*). The Sc substitution ratio was set to about 3 at % (Sc_0.09_Ti_2.91_O_5_). That is, 1 of 36 Ti atoms was substituted by an Sc atom in the supercells. The differential energy of λ and β phase was calculated (Δ*G* = *G*_λ_ − *G*_β_). The calculated Δ*G* of Ti_3_O_5_ and Sc-substituted Ti_3_O_5_ are shown in Fig. 4. Ti_3_O_5_ shows Δ*G* = 0 at 575°C (848 K), which is the crossover temperature of the calculated free energies corresponding to the phase transition temperature (*29*). Sc-substituted Ti_3_O_5_ showed Δ*G* = 0 at 341°C (614 K). The normalized temperature in Fig. 4 was set at 575°C (848 K), which is the crossover temperature of the free energies of Ti_3_O_5_. The crossover temperature of Sc-substituted Ti_3_O_5_ decreased about 27.6% from the temperature of Ti_3_O_5_. This temperature decrease ratio agreed well with the experimentally obtained decrease ratio of 27.7% calculated from the phase transition temperature of 470 K (197°C) reported in λ-Ti_3_O_5_ (*16*) and 340 K (67°C) measured in λ-Sc_0.09_Ti_2.91_O_5_. The calculated crossover temperature was overestimated compared with the phase transition temperature, which was likely because the magnetic interaction was not taken into account during the phonon calculations. The formation energies corresponding to the Gibbs free energies (*G*) at 0 K were −2362.47 eV (λ-Ti_3_O_5_), −2376.44 eV (Sc-substituted λ-Ti_3_O_5_), −2372.45 eV (β-Ti_3_O_5_), and −2381.65 eV (Sc-substituted β-Ti_3_O_5_).

### Thermodynamic analysis

In the SD model calculations, the γ value depends on the temperature and pressure (i.e., γ = γ*_a_* + γ*_b_T* + γ*_c_P*). From the DSC measurement result, the Δ*H* value was 4.0 kJ mol^−1^, and the Δ*S* value was 11.7 J K^−1^ mol^−1^. When the parameters of γ were set as γ*_a_* = 7 kJ mol^−1^, γ*_b_* = −1.2 J K^−1^ mol^−1^, and γ*_c_* = −0.37 J MPa^−1^ mol^−1^, the SD model calculations reproduced the long-term heat storage and pressure-induced phase transition, as shown in Fig. 5.

## Supplementary Material

aaz5264_SM.pdf

## SUPPLEMENTARY MATERIALS

Supplementary material for this article is available at http://advances.sciencemag.org/cgi/content/full/6/27/eaaz5264/DC1
