# Supplementary material for: Long-term heat-storage ceramics absorbing thermal energy from hot water
Source: Sci Adv. 2020 Jul 1;6(27):eaaz5264. doi: 10.1126/sciadv.aaz5264 (PMC7329333; doi:10.1126/sciadv.aaz5264)
Supplement: aaz5264_SM.pdf [file aaz5264_SM.pdf]

Science Advances
AAAS

advances.sciencemag.org/cgi/content/full/6/27/eaaz5264/DC1

Supplementary Materials for

Long-term heat-storage ceramics absorbing thermal energy from hot water

Yoshitaka Nakamura*, Yuki Sakai, Masaki Azuma, Shin-ichi Ohkoshi*

*Corresponding author. Email: nakamura.yoshi-taka@jp.panasonic.com (Y.N.); ohkoshi@chem.s.u-tokyo.ac.jp (S.O.)

Published 1 July 2020, *Sci. Adv.* **6**, eaaz5264 (2020)
DOI: 10.1126/sciadv.aaz5264

This PDF file includes:

Figs. S1 to S9
Table S1

SUPPLEMENTARY MATERIALS

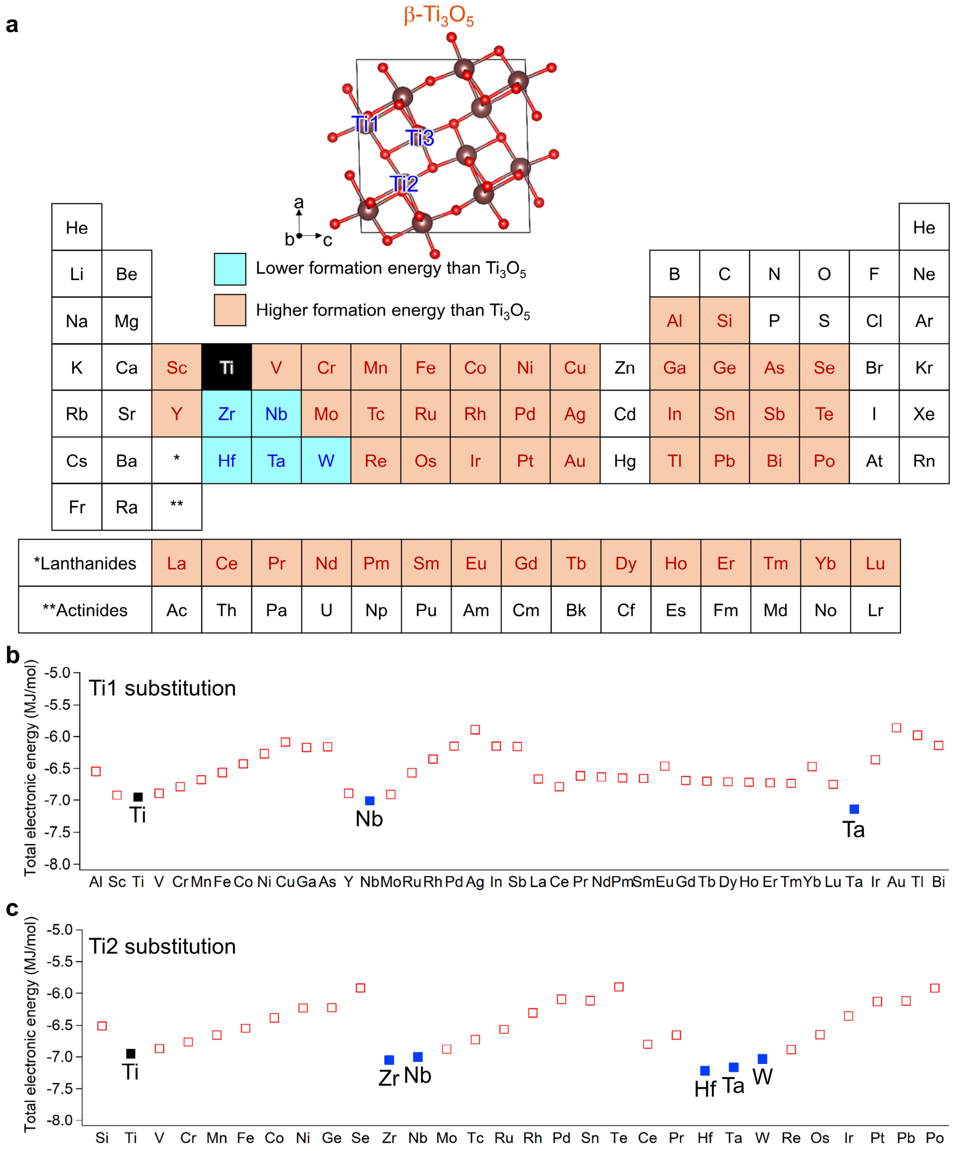

Fig. S1-1. First-principles calculations of formation energies. a Periodic table coloured by the total electronic energies of β-Ti_3O_5 with an elemental substitution. Blue elements are those where substituted β-Ti_3O_5 shows a lower formation energy than that of pure β-Ti_3O_5. Orange elements are those where substituted β-Ti_3O_5 shows a higher formation energy. **b** Calculated total electronic energies of β-$A_xTi_{3-x}O_5$ (A = trivalent elements) and **c** β-$B_xTi_{3-x}O_5$ (B = tetravalent elements) in order of the atomic number. One of the three Ti sites in β-Ti_3O_5 is substituted by a coloured-element for the first-principles calculations. Element A in β-$A_xTi_{3-x}O_5$ is substituted into the Ti1 site. Element B in β-$B_xTi_{3-x}O_5$ is substituted into the Ti2 site. Unlike Sc-substituted λ-Ti_3O_5, which has a lower formation energy than pure λ-Ti_3O_5, Sc-substituted β-Ti_3O_5 shows a higher formation energy.

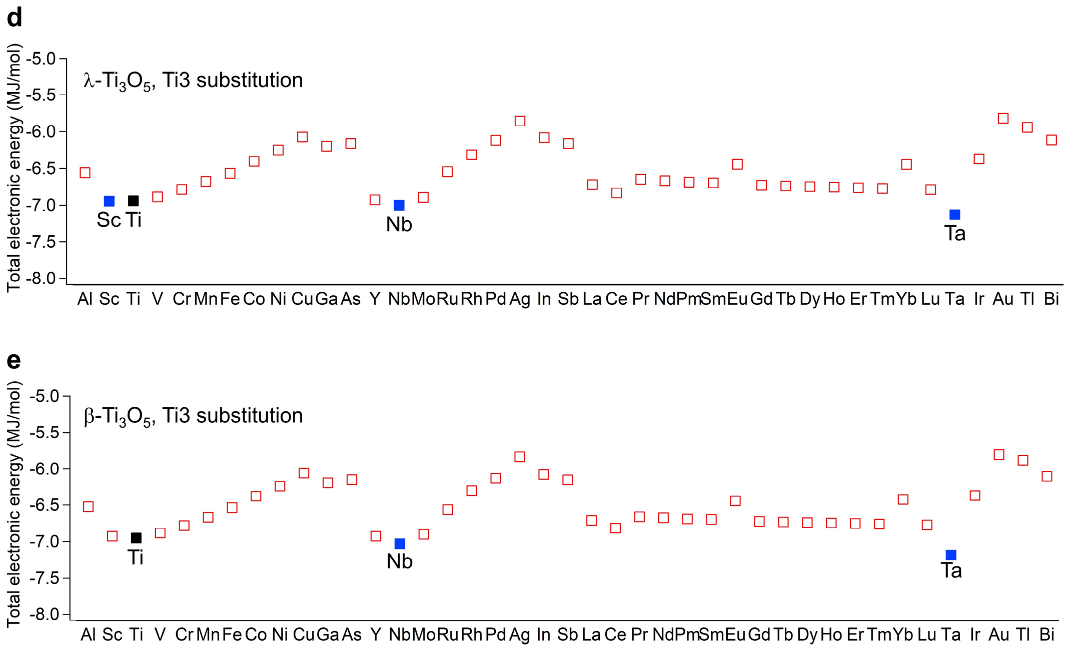

Fig. S1-2. First-principles calculations of formation energies. Calculated total electronic energies of **d** λ-$A_xTi_{3-x}O_5$ and **e** β-$A_xTi_{3-x}O_5$ (A = trivalent elements) in order of atomic number. Ti3 site in λ- and β-Ti_3O_5 is substituted by a coloured-element (shown in Figure 1a and Fig. S1-1a) for the first-principles calculations. λ-$A_xTi_{3-x}O_5$ (A = Sc, Nb, and Ta) and β-$A_xTi_{3-x}O_5$ (A = Nb and Ta) show lower formation energies than Ti_3O_5. These calculation results are the same as the results for λ- and β-Ti_3O_5 with Ti1 site substitution (shown in Figure 1b and Fig. S1-1b).

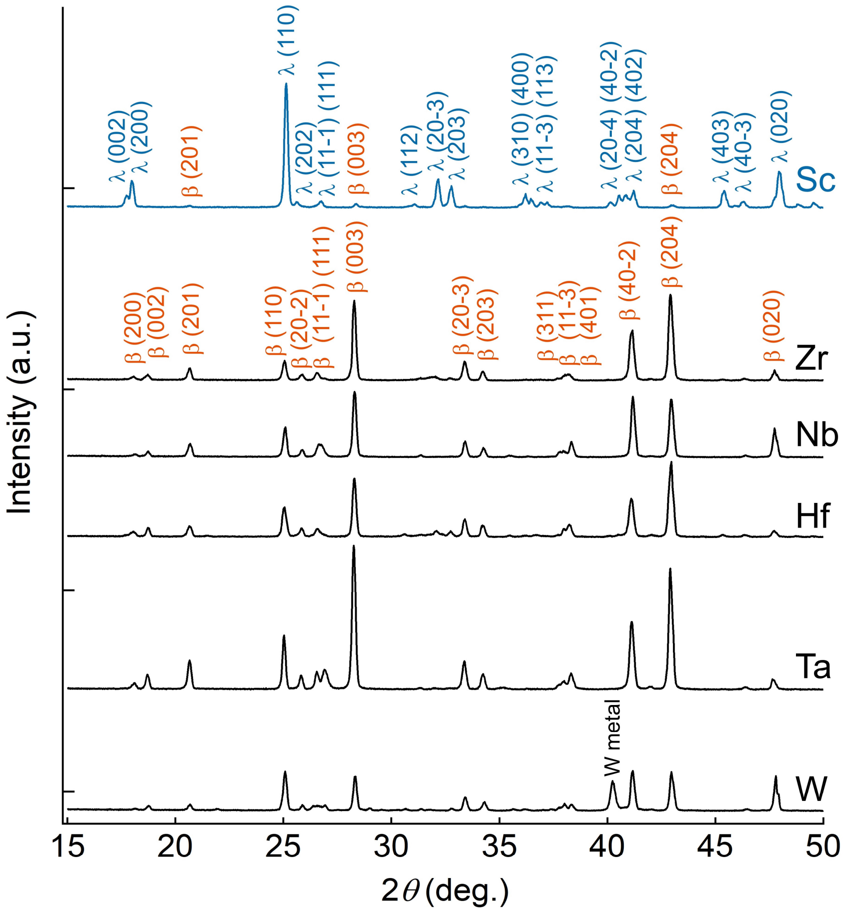

Fig. S2. X-ray diffraction (XRD) patterns of Ti_3O_5 substituted by 3 at% of Sc, Zr, Nb, Hf, Ta, and W. Only Sc-substituted Ti_3O_5 shows the λ-phase. All others show the β-phase only.

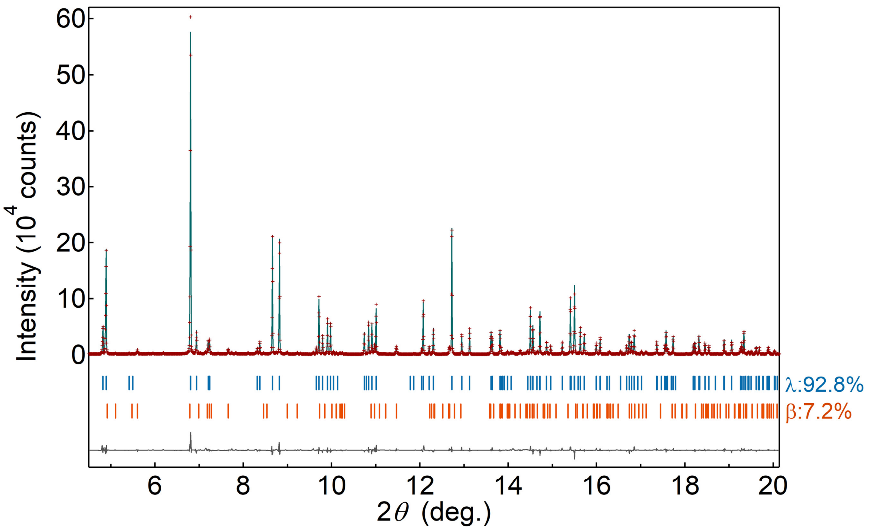

Polymorph	λ-$Sc_{0.09}Ti_{2.91}O_5$
Crystal system	Monoclinic
Space group	*C*2/*m* (No.12)
a (Å)	9.84195 (4)
b (Å)	3.79151 (1)
c (Å)	9.98618 (4)
β (°)	91.1207 (3)
V (Å^3)	372.572 (3)
Z	4
R_{wp} (%)	10.560
S	8.2877

Polymorph	β-$Sc_{0.09}Ti_{2.91}O_5$
Crystal system	Monoclinic
Space group	*C*2/*m* (No.12)
a (Å)	9.7930 (4)
b (Å)	3.8064 (14)
c (Å)	9.4375 (4)
β (°)	91.5611 (3)
V (Å^3)	351.66 (2)
Z	4
R_{wp} (%)	10.560
S	8.2877

	g	*x/a*	*y/b*	*z/c*	*B* (Å^2)
Ti(1)	0.97	0.6304 (2)	0	0.0531 (19)	0.734
Sc(1)	0.03	0.6304	0	0.0531	0.734
Ti(2)	0.97	0.3035 (18)	0	0.2466 (2)	1.031
Sc(2)	0.03	0.3035	0	0.2466	1.031
Ti(3)	0.97	0.6352 (19)	0	0.4343 (18)	0.725
Sc(3)	0.03	0.6352	0	0.4343	0.725
O(1)	1	0.4487 (6)	0	0.3850 (5)	0.200
O(2)	1	0.1795 (6)	0	0.0644 (6)	1.742
O(3)	1	0.7379 (6)	0	0.2467 (6)	0.200
O(4)	1	0.5479 (6)	0	0.8715 (5)	0.200
O(5)	1	0.1897 (6)	0	0.4265 (5)	0.200

	g	*x/a*	*y/b*	*z/c*	*B* (Å^2)
Ti(1)	0.97	0.126 (17)	0	0.048 (3)	2.000
Sc(1)	0.03	0.126	0	0.048	2.000
Ti(2)	0.97	0.775 (19)	0	0.269 (18)	2.000
Sc(2)	0.03	0.775	0	0.269	2.000
Ti(3)	0.97	0.053 (2)	0	0.355 (3)	2.000
Sc(3)	0.03	0.053	0	0.355	2.000
O(1)	1	0.675 (6)	0	0.043 (5)	0.200
O(2)	1	0.239 (6)	0	0.244 (5)	0.200
O(3)	1	0.605 (5)	0	0.364 (6)	2.000
O(4)	1	0.937 (5)	0	0.153 (8)	0.200
O(5)	1	0.872 (5)	0	0.458 (7)	0.200

Fig. S3. Rietveld analysis of the SXRD data of $Sc_{0.09}Ti_{2.91}O_5$. The SXRD pattern was measured at ambient temperature and pressure (λ = 0.420111 Å). Refined phase fractions are plotted in Figure 3b, and the same figure appears in Figure 2a. Refined structural parameters of $Sc_{0.09}Ti_{2.91}O_5$ at ambient temperature and pressure. Occupation factors of Ti and Sc were fixed at the nominal ratio because these are indistinguishable by X-ray.

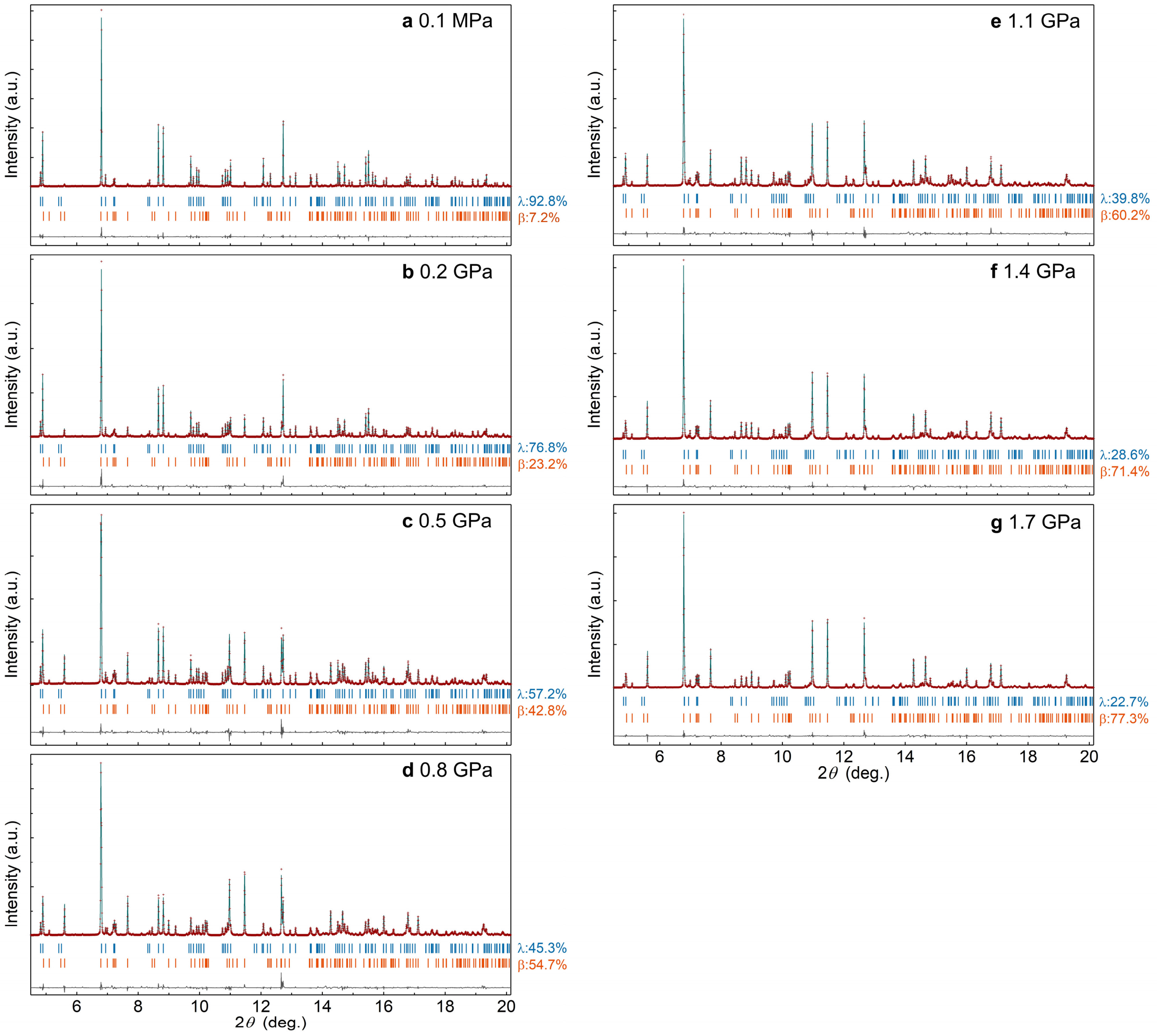

Fig. S4. SXRD patterns of pressure-applied $Sc_{0.09}Ti_{2.91}O_5$. Rietveld analyses of the SXRD data of $Sc_{0.09}Ti_{2.91}O_5$ measured at **a** ambient temperature and pressure, after compression at **b** 0.2 GPa, **c** 0.5 GPa, **d** 0.8 GPa, **e** 1.1 GPa, **f** 1.4 GPa, and **g** 1.7GPa (λ = 0.420111 Å). Refined phase fractions are plotted in Figure 3b.

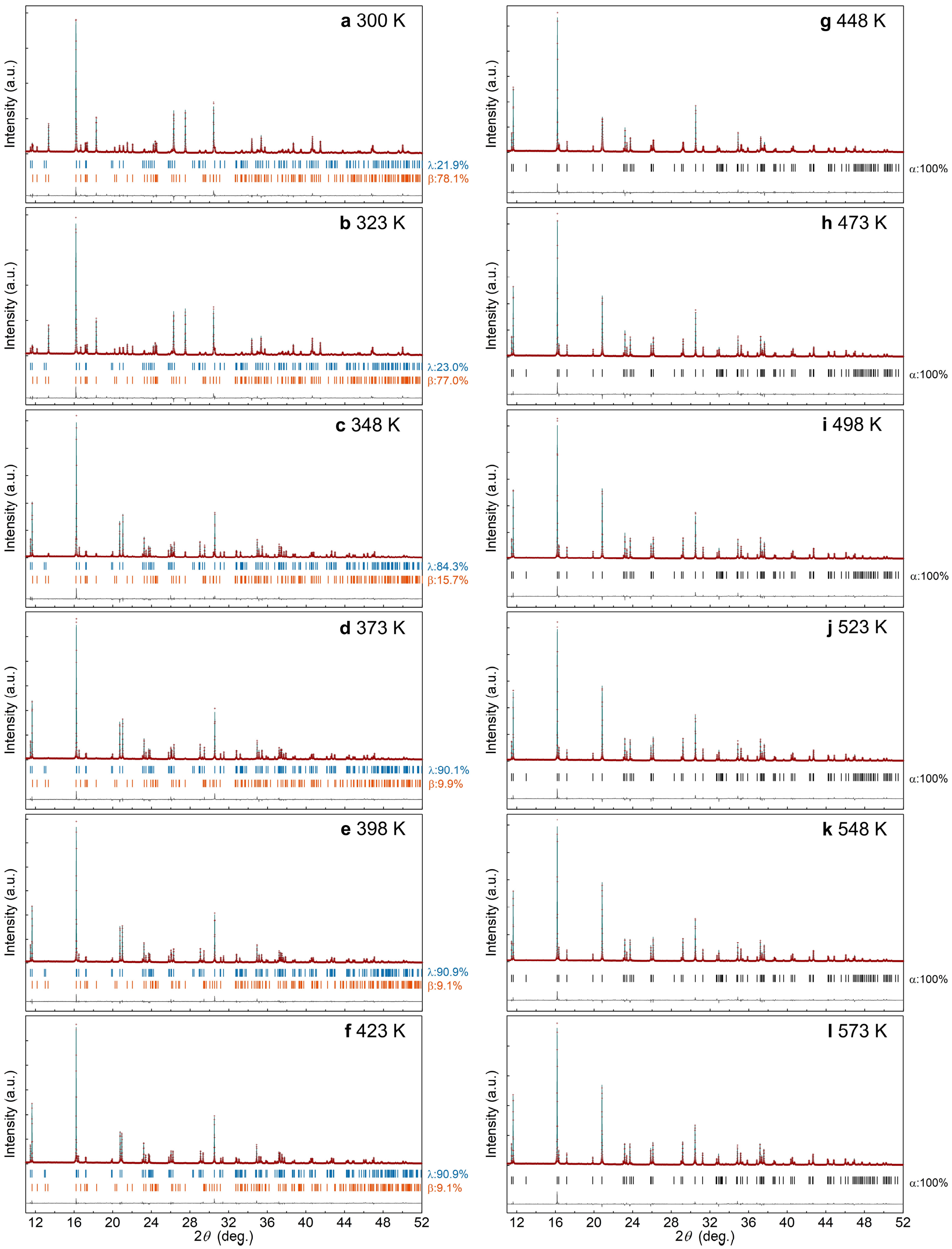

Fig. S5-1. Temperature-variable SXRD patterns of $Sc_{0.09}Ti_{2.91}O_5$. Rietveld analyses of the SXRD data of $Sc_{0.09}Ti_{2.91}O_5$ measured at **a** 300 K, **b** 323 K, **c** 348 K, **d** 373 K, **e** 398 K, **f** 423 K, **g** 448 K, **h** 473 K, **i** 498 K, **j** 523 K, **k** 548 K, and **l** 573 K (λ = 0.999255 Å). Refined phase fractions and lattice parameters are plotted in Figure S6.

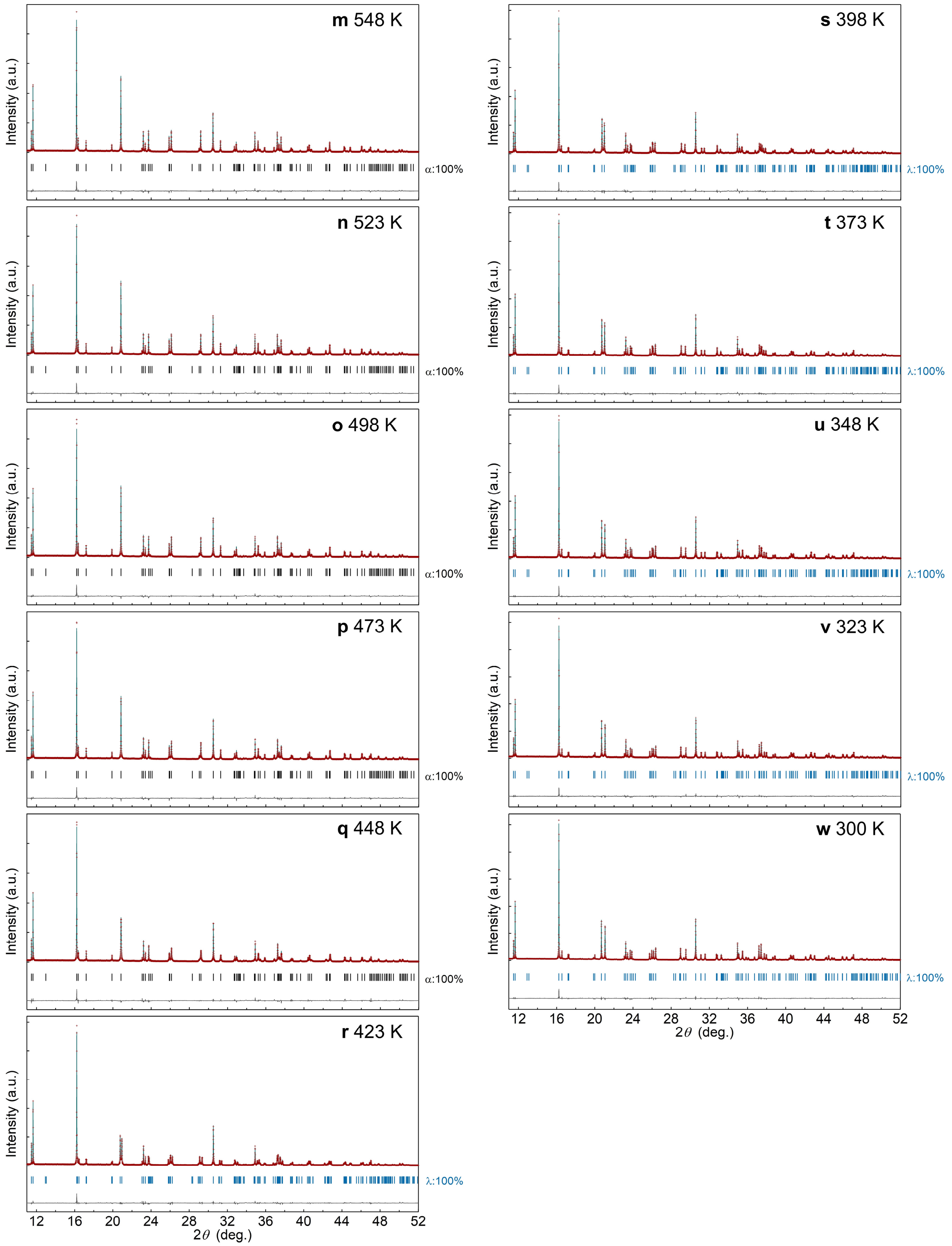

Fig. S5-2. Temperature-variable SXRD patterns of $Sc_{0.09}Ti_{2.91}O_5$. Rietveld analyses of the SXRD data of $Sc_{0.09}Ti_{2.91}O_5$ measured at **m** 548 K, **n** 523 K, **o** 498 K, **p** 473 K, **q** 448 K, **r** 423 K, **s** 398 K, **t** 373 K, **u** 348 K, **v** 323 K, and **w** 300 K (λ = 0.999255 Å). Refined phase fractions and lattice parameters are plotted in Figure S6.

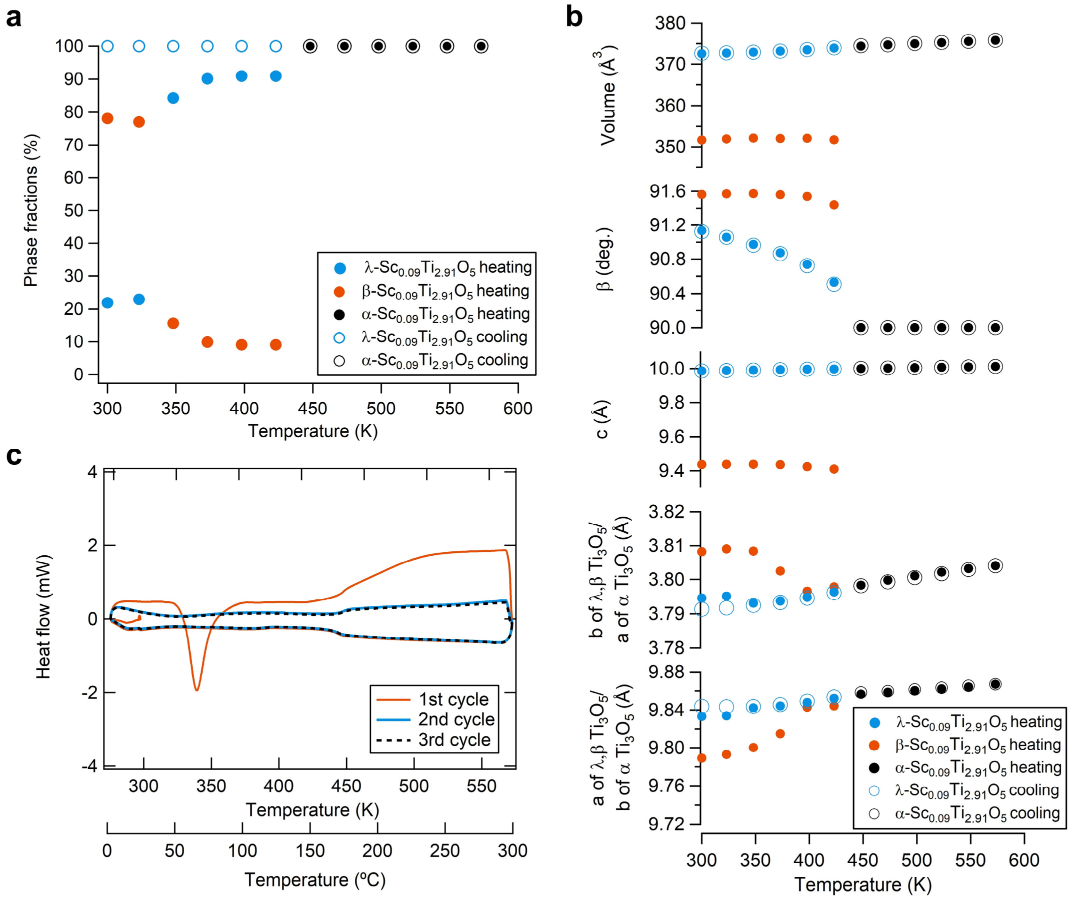

Fig. S6. Temperature dependence of the crystal structure and heat-absorption process. a Temperature dependence of the phase fractions of $Sc_{0.09}Ti_{2.91}O_5$ calculated from the SXRD data. **b** Lattice parameters and unit cell volume of $Sc_{0.09}Ti_{2.91}O_5$. **c** DSC charts of $Sc_{0.09}Ti_{2.91}O_5$ measured at 0–300 °C (273–573 K) in three cycles. The first heating-cooling loop shows an endothermic reaction at 67 °C (340 K), but no reaction for the second and third cycles. This result agrees well with the phase transition from the β- to λ-phase observed in the variable temperature SXRD measurements (Fig. 3c) and the absence of a transition back to the β-phase upon cooling. DSC curves have a shoulder around 167–177 °C (440–450 K), corresponding to the transition from the λ- to α-phase observed in the variable temperature SXRD measurements (Fig. 3c). Measured heat absorption mass is 58 kJ L^{-1}, which means that 77.3% of the β-phase transforms to the λ-phase. Conversion of the λ- and β-phases, or the heat absorption mass of $Sc_{0.09}Ti_{2.91}O_5$, is 75 kJ L^{-1}.

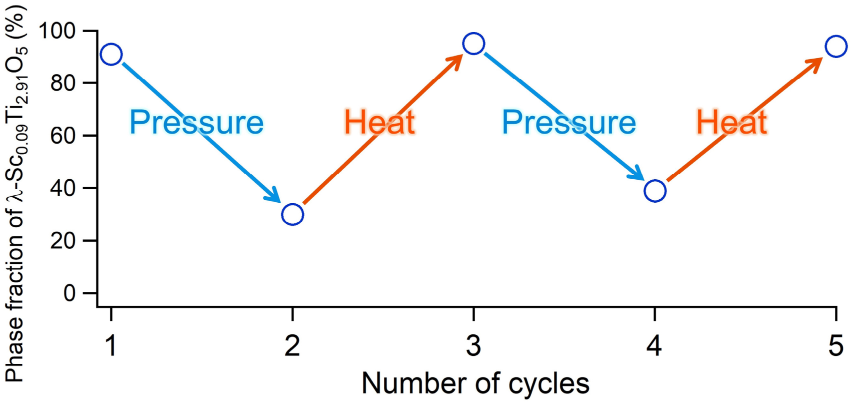

Fig. S7. Repeatability of the pressure- and heat-induced phase transition. In the pressure application process (blue lines), the λ-$Sc_{0.09}Ti_{2.91}O_5$ sample is compressed at 1.7 GPa at room temperature and the phase fraction is calculated from the laboratory XRD data of the pressure released sample. In the heating process (red lines), the pressure released sample is heated to 473 K (200 °C) and cooled to room temperature. In these pressure applications and heating cycles, the phase fraction of λ-$Sc_{0.09}Ti_{2.91}O_5$ shows a good repeatability.

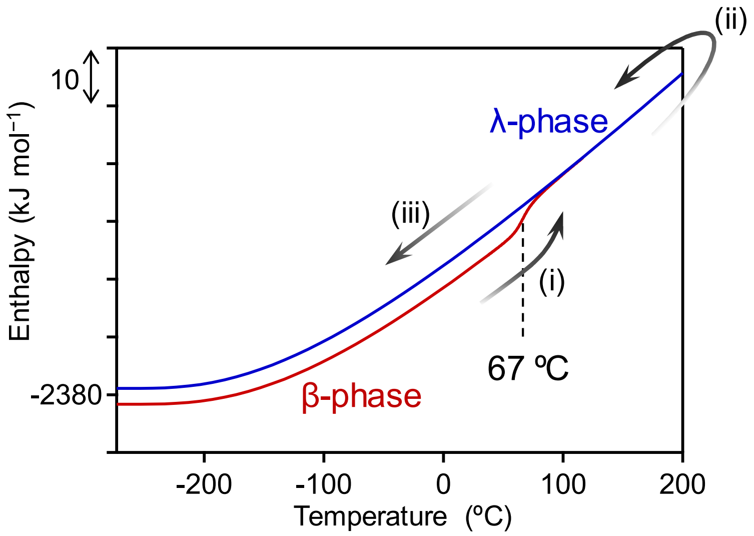

Fig. S8. Mechanism of long-term heat storage. Temperature dependence of the enthalpy for the λ-phase (blue line) and β-phase (red line) of $Sc_{0.09}Ti_{2.91}O_5$ obtained from the temperature dependence of the enthalpy from the phonon mode calculation and the transition enthalpy from the DSC measurement. Curves are offset to the formation energy of the β-phase at 0 K, which is evaluated by first-principles calculations. (i) Pressure-produced β-phase accumulates the heat energy and transforms to λ-phase at 67 °C. (ii) Temperature of the λ-phase is increased and then decreased again, and (iii) the λ-phase is maintained even at low temperatures.

The transformation energy efficiency (e) can be described by

$$\underbrace{H_{\text{output}}}_{\substack{\text{Extractable}\\ \text{enthalpy}}} = \underbrace{\Delta H}_{\substack{\text{Accumulated}\\ \text{heat energy}\\ \text{at 67 °C}}} - \underbrace{(C_{\lambda} - C_{\beta})(T_{\text{p}} - T_0)}_{\substack{\text{Energy loss from}\\ \text{heat capacity difference}\\ \text{between } \lambda \text{ and } \beta}} - \underbrace{C_{\beta}(T_1 - T_0)}_{\substack{\text{Sensible loss from warming}\\ \text{of the material itself}}}$$

$$e = H_{\text{output}} / \Delta H$$

where H_{output} is the maximum heat to be taken out from the material, ΔH is the accumulated heat energy, T_{p} is the phase transition temperature (= 67 °C), T_0 is the heat release temperature, T_1 is the final temperature after warming, C_{λ} is the heat capacity of the λ-phase, and C_{β} is the heat capacity of the β-phase. Based on the temperature dependence of the enthalpy for the λ-phase and β-phase obtained by first-principles calculations and DSC measurement, the e value is obtained.

Table S1. Calculated e values of $Sc_xTi_{3-x}O_5$ for each ($T_1 - T_0$) value when T_0 = 15 °C.

$T_1 - T_0$ (K)	1	2	3	4	5
e (%)	93	89	85	81	77

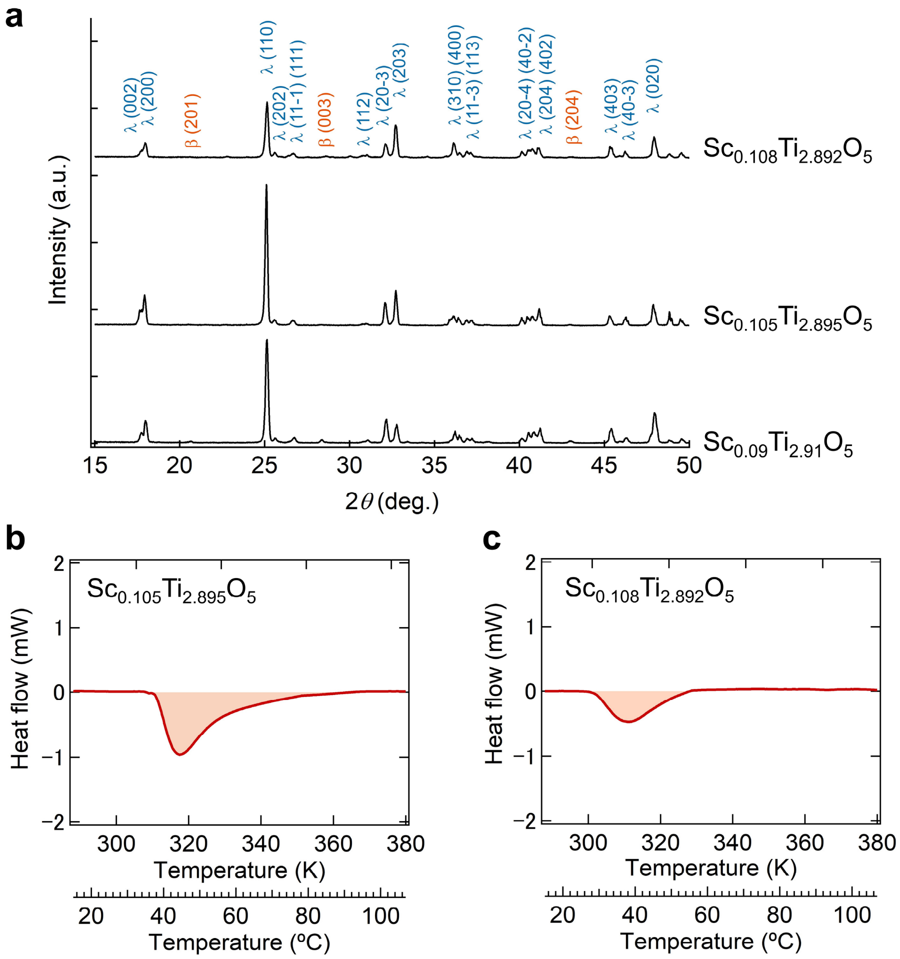

Fig. S9. X-ray diffraction (XRD) patterns and heat-storage property of Sc-substituted Ti_3O_5. a XRD patterns of $Sc_{0.09}Ti_{2.91}O_5$, $Sc_{0.105}Ti_{2.895}O_5$, and $Sc_{0.108}Ti_{2.892}O_5$ synthesised by an arc melting method. DSC charts of **b** $Sc_{0.105}Ti_{2.895}O_5$ and **c** $Sc_{0.108}Ti_{2.892}O_5$. Samples are compressed at 1.7 GPa before the measurement. $Sc_{0.105}Ti_{2.895}O_5$ shows heat-absorption with an absorption peak at 45 °C (318 K). λ-$Sc_{0.108}Ti_{2.892}O_5$ shows heat absorption with an absorption peak at 38 °C (311 K).
